# Pediatric Bacterial Meningitis Surveillance in Nigeria From 2010 to 2016, Prior to and During the Phased Introduction of the 10-Valent Pneumococcal Conjugate Vaccine

**DOI:** 10.1093/cid/ciz474

**Published:** 2019-09-05

**Authors:** Beckie N Tagbo, Rowan E Bancroft, Iretiola Fajolu, Mohammed B Abdulkadir, Muhammad F Bashir, Olusola P Okunola, Ayodeji H Isiaka, Namadi M Lawal, Benedict O Edelu, Ngozi Onyejiaka, Chinonyerem J Ihuoma, Florence Ndu, Uchenna C Ozumba, Frances Udeinya, Folasade Ogunsola, Aishat O Saka, Abayomi Fadeyi, Sunday A Aderibigbe, Jimoh Abdulraheem, Adamu G Yusuf, Peter Sylvanus Ndow, Philomena Ogbogu, Chinomnso Kanu, Velly Emina, Olajumoke J Makinwa, Florian Gehre, Kabir Yusuf, Fiona Braka, Jason M Mwenda, Johnson M Ticha, Dorothy Nwodo, Archibald Worwui, Joseph N Biey, Brenda A Kwambana-Adams, Martin Antonio

**Affiliations:** 1 Institute of Child Health, University of Nigeria Teaching Hospital, Ituku-Ozalla; 2 Department of Paediatrics University of Nigeria Teaching Hospital Ituku-Ozalla, Enugu State; 3 World Health Organization (WHO) Collaborating Centre for New Vaccines Surveillance, Medical Research Council Unit The Gambia at London School of Hygiene & Tropical Medicine, Banjul; 4 Department of Paediatrics, Lagos University Teaching Hospital; 5 Department of Paediatrics, College of Medicine, University of Lagos; 6 Department of Paediatrics and Child Health, University of Ilorin Teaching Hospital; 7 Department of Paediatrics, Abubakar Tafawa Balewa University Teaching Hospital, Bauchi; 8 Department of Child Health, University of Benin Teaching Hospital; 9 WHO Country office, Abuja; 10 Department of Disease Control and Immunization, National Primary Health Care Development Agency, Abuja; 11 Department of Medical Microbiology and Parasitology, Lagos University Teaching Hospital; 12 Department of Microbiology, University of Nigeria Teaching Hospital, Ituku-Ozalla, Enugu State; 13 Mother of Christ Specialist Hospital Enugu; 14 Department of Medical Microbiology and Parasitology, University of Ilorin Teaching Hospital, Kwara; 15 Department of Epidemiology and Community Health, University of Ilorin Teaching Hospital, Kwara; 16 Medical Microbiology Department, Abubakar Tafawa Balewa University Teaching Hospital, Bauchi; 17 Department of Medical Microbiology, University of Benin Teaching Hospital; 18 Department of Community Health, University of Benin Teaching Hospital; 19 Department of Community Health and Primary Care, Lagos University Teaching Hospital, Nigeria; 20 Department of Infectious Disease Epidemiology, Bernhard-Nocht-Institute for Tropical Medicine, Hamburg, Germany; 21 WHO, Nigeria EPI Cluster Lead; 22 WHO Regional Office for Africa WHO/AFRO, Republic of Congo, Brazzaville; 23 Microbiology and Infection Unit, Warwick Medical School, University of Warwick, Coventry, United Kingdom

**Keywords:** pediatric, meningitis, Nigeria, neumococcus, meningococcus

## Abstract

**Background:**

Historically, Nigeria has experienced large bacterial meningitis outbreaks with high mortality in children. *Streptococcus pneumoniae* (pneumococcus)*, Neisseria meningitidis* (meningococcus), and *Haemophilus influenzae* are major causes of this invasive disease. In collaboration with the World Health Organization, we conducted longitudinal surveillance in sentinel hospitals within Nigeria to establish the burden of pediatric bacterial meningitis (PBM).

**Methods:**

From 2010 to 2016, cerebrospinal fluid was collected from children <5 years of age, admitted to 5 sentinel hospitals in 5 Nigerian states. Microbiological and latex agglutination techniques were performed to detect the presence of pneumococcus, meningococcus, *and H. influenzae.* Species-specific polymerase chain reaction and serotyping/grouping were conducted to determine specific causative agents of PBM.

**Results:**

A total of 5134 children with suspected meningitis were enrolled at the participating hospitals; of these 153 (2.9%) were confirmed PBM cases. The mortality rate for those infected was 15.0% (23/153). The dominant pathogen was pneumococcus (46.4%: 71/153) followed by meningococcus (34.6%: 53/153) and *H. influenzae* (19.0%: 29/153). Nearly half the pneumococcal meningitis cases successfully serotyped (46.4%: 13/28) were caused by serotypes that are included in the 10-valent pneumococcal conjugate vaccine. The most prevalent meningococcal and *H. influenzae* strains were serogroup W and serotype b, respectively.

**Conclusions:**

Vaccine-type bacterial meningitis continues to be common among children <5 years in Nigeria. Challenges with vaccine introduction and coverage may explain some of these finding. Continued surveillance is needed to determine the distribution of serotypes/groups of meningeal pathogens across Nigeria and help inform and sustain vaccination policies in the country.

## Teaser

*Streptococcus pneumoniae* was the predominant pathogen responsible pediatric bacterial meningitis (46.4%: 71/153) during longitudinal sentinel surveillance within Nigeria, from 2010 to 2016. However, with the introduction of PCV10 across Nigeria, it is hoped that rates of pneumococcal meningitis will decline.

Acute bacterial meningitis is a much-dreaded infectious disease in children and a major cause of morbidity and mortality [1, 2]. The incidence and case-fatality rates for this invasive bacterial disease vary by region, country, causative-agent, and age-group affected, but without adequate treatment, fatalities can be as high as 70% [1, 3]. The African meningitis belt is the most significantly affected area worldwide, with an estimated 400 million people at-risk annually. Bacterial meningitis epidemics have occurred frequently across this region for over a century: one of the largest occurred in Nigeria during 1996, with over 109 000 cases and 11 000 deaths [4]. Subsequently, there have been several major outbreaks within Nigeria, over wide geographical areas, most notably in 2009 and 2015 [4, 5]. In December 2016, the Nigeria Centre for Disease Control (NCDC) received reports of a meningitis outbreak. By May 2017, there were 13 420 suspected cases and 1069 deaths (8%), of which 47% were children in the 5–14 year age group [6].

The leading causes of bacterial meningitis are *Neisseria meningitidis* (meningococcus), *Streptococcus pneumoniae* (pneumococcus), and *Haemophilus influenzae*; which are usually carried asymptomatically in the nasopharynx and transmitted through respiratory droplets [1]. Normally, the pathogen responsible for epidemics is the *Neisseria* species, whereas pneumococcus and *H. influ*enzae are endemic throughout the year [4, 7, 8]. In 2008, the World Health Organization (WHO) established the Global Invasive Bacterial-Vaccine Preventable Diseases (IB-VPD) network building on regional surveillance networks. This global surveillance network encompasses 100 sentinel hospital laboratories, aiming to estimate the burden of bacterial meningitis and characterize circulating bacterium within member countries [2, 9]. As part of this, the Medical Research Council Unit The Gambia at the London School of Hygiene and Tropical Medicine (MRCG at LSHTM), a WHO Collaborating Center for New Vaccines Surveillance (WHO CC NVs), provides laboratory support for pediatric bacterial meningitis (PBM) surveillance in 10 countries across West Africa, including Nigeria.

Nigeria is the most populous country within Africa, with an estimated 193 million people living in 36 states [10]. The national human immunodeficiency virus (HIV) prevalence among adults aged 15–49 year in Nigeria is 1.4%; however, this varies between states with 5.6% estimated in the southern Akwa Ibom State and 0.3% in the northwestern Katsina State [11, 12]. In 2012, the Nigerian Expanded Program on Immunization (EPI) replaced the diphtheria-tetanus-pertussis and hepatitis B vaccines with a pentavalent vaccine that included *H. influenzae* type b (Hib) and underwent a phased introduction [13]. Additionally, meningococcal A conjugate vaccine (MenAfriVac) campaigns were carried out from 2011 to 2014 in 19 states in Northern Nigeria. The 2017 outbreak was caused by meningococcal serogroup C; thus, a vaccine with longevity against this strain is required [14]. A phased introduction of the 10-valent pneumococcal conjugate vaccine (PCV10) throughout Nigeria, targeting 10 invasive pneumococcal serotypes, commenced in December 2014 and concluded in October 2016. However, the 2017 WHO and United Nations Children’s Fund (UNICEF) national immunization coverage estimates suggest that coverage rates for children who received 3 doses of PCV10 and Hib-containing vaccines remain low, at 36% and 42%, respectively [15]. As part of the IB-VPD network, longitudinal surveillance was established in 2010 within 5 sentinel hospitals in 5 Nigerian states. We have conducted analysis to estimate the prevalence of bacterial meningitis in children <5 years of age and determine the distribution of the causative pathogens from 2010 to 2016.

## METHODS

### Surveillance Sites

Surveillance of children <5 years admitted to sentinel hospitals with suspected meningitis, was carried out for 7 years. PBM surveillance started in Lagos University Teaching Hospital (Lagos State) in 2010, followed by University of Nigeria Teaching Hospital (Enugu State) in 2011. In the following year, 2 more sites were included: Abubakar Tafawa Balewa University Teaching Hospital (Bauchi State) and University of Ilorin Teaching Hospital (Kwara State). Finally, in 2013, University of Benin Teaching Hospital (Edo State) joined surveillance. Therefore, surveillance was conducted in 5 states with a combined estimated population of approximately 32 million people.

### Case Enrollment

Hospitalized children aged 0–59 months (<5 years) with features of suspected meningitis: rapid onset of fever with axillary or rectal temperatures of >38°C and >38.5°C, combined with any of the following symptoms: impaired consciousness, meningismus (stiff neck), photophobia, bulging fontanelle (infants), and convulsions were enrolled in the surveillance [16]. A lumbar puncture (LP) was performed for routine diagnostic tests, and cerebrospinal fluid (CSF) was collected.

### CSF Analysis

CSF samples were processed following the WHO standard operating procedure [16]. An aliquot of CSF was centrifuged, and the deposit inoculated onto Columbia blood and chocolate agar plates and incubated overnight. Isolates of the target pathogens were identified using the optochin test (5 μg optochin disk; Oxoid, Basingstoke, UK) for pneumococcus and analytical profile index (API NH; Biomerieux, Basingstoke, UK) for meningococcus and *H. influenzae.* The remaining centrifuged pellet was used to prepare smears for Gram staining. Using the supernatant, latex agglutination was performed with the Pastorex meningitis kit (Biorad, Watford, UK), for the detection of pneumococcus, Hib and meningococcus groups A, B, C, Y, and W antigens. When possible, CSF was used to detect the presence of pneumococcus using the BINAX® NOW kit (Alere Inc., Waltham, MA, USA). A white blood cell (WBC) count was conducted along with CSF protein and glucose analysis using trichloroacetic acid turbidimetric and glucose oxidase methods [17, 18].

### Molecular Analysis

CSF samples were transported to the MRCG at LSHTM according to International Air Transport Association (IATA) regulations [19]. Species-specific quantitative polymerase chain reaction (qPCR) assays for detection of pneumococcus, meningococcus, and *H. influenzae* were performed, using the autolysin gene (*lytA*), Cu, Zn superoxide dismutase gene (*sodC*) and protein D encoding gene (*hpd*), respectively, as described elsewhere [20]. For amplification, samples were heated at 95°C for 10 minutes, followed by 45 cycles of 95°C for 15 seconds and 60°C for 1 minute. Cycle threshold (CT) values of ≤36 were considered positive results.

### Serotyping and Serogrouping

Meningococcus and *H. influenzae* serogrouping/typing was conducted using direct qPCR. Meningococcal gene targets were: *sacB, synD, synE, synG, xcbB, synF* for serogroups A, B, C, W, X, and Y, respectively. Additionally, for *H. influenzae* gene targets included *acsB, bcsB, ccsD, dscE, ecsH, bexD* for serotypes Hia, Hib, Hic, Hid, Hie, and Hif, respectively. CT values of ≤32 were considered positive [21]. For pneumococcus, nucleic acid extraction using Qiagen DNA Mini-kit was performed. Purified DNA underwent sequential triplex qPCR for detection of 21 capsular serotypes as described elsewhere [22]. Nontypeable pneumococci, with CT values ≤32 by qPCR, were further subjected to conventional multiplex serotyping PCR assays.

### Statistical Analysis

Data were collected at the sentinel hospitals using a standardized WHO Regional Office for Africa (Afro) PBM network case report form. Information recorded included, patient demographics, clinical symptoms, vaccination history, laboratory information (CSF microscopy, bacteriological tests, genotyping), and outcome at discharge. Data were subsequently put into a WHO Epi Info-based customized new-vaccine surveillance data module. Data cleaning and analysis were first performed at the sentinel site level before being sent to the national and regional WHO data managers. At the national level, data from sites were merged, cleaned, analyzed, and interpreted. Merged data were then sent to WHO Afro data managers, and feedback was provided to sites every 3–6 months. WHO Afro also sent regional data to WHO Global data managers. For presentation here, data were analyzed using GraphPad Prism 8.1.1; percentages, proportion, means, and standard deviations were calculated as appropriate and presented as prose, tables, and figures.

Ethical approval was not a requirement in Nigeria for routine meningitis surveillance including drug susceptibility testing of collected isolates as this is approved within the routine diagnostic algorithm at the Ministry of Health. However, informed consent was sought from caregivers of the surveillance participants. Additionally, the surveillance received overarching ethical approval (SCC1188) by the joint MRC/The Gambia Government ethics board that allowed the analysis of collected West African isolates at MRC Unit, The Gambia.

## RESULTS

### Demographic and Clinical Characteristics

Details of the demographic characteristics of the children enrolled in surveillance are shown in Table 1. A total of 5134 children <5 years of age with suspected bacterial meningitis were enrolled at the sentinel hospitals from 2010 to 2016. Of these, 5008 (97.5%: 5008/5134) children had CSF samples collected, which then underwent diagnostic testing. Overall, 57.8% (2969/5134) of patients were male and the median age of patients was 22 months (interquartile range: 1–23). The largest proportion of meningitis cases were reported in children from the youngest age group, 0–11 months, with 3014 (58.7%: 3014/5134) suspected cases. Unfortunately, 278 patients with suspected meningitis died in hospital resulting in a case fatality of 5.4% (278/5134).

**Table 1.** Summary of Demographic Characteristics of Study Population

		Total	Bauchi^a^	Lagos^b^	Edo^c^	Kwara^d^	Enugu^e^
Characteristic	Category	n (%)	n (%)	n (%)	n (%)	n (%)	n (%)
Age	0–11 m	3014 (58.7)	230 (32.4)	1473 (72.3)	588 (67.1)	222 (37.1)	501 (54.9)
	12–23 m	798 (15.5)	120 (16.9)	222 (10.9)	120 (13.7)	127 (21.2)	209 (22.9)
	24–59 m	1234 (24.0)	358 (50.4)	264 (13.0)	164 (18.7)	247 (41.3)	201 (22.0)
	Unknown	88 (1.7)	2 (0.3)	79 (3.9)	4 (0.5)	2 (0.3)	1 (0.1)
Sex	Female	2147 (41.8)	300 (42.3)	843 (41.4)	356 (40.6)	269 (45.0)	379 (41.6)
	Male	2969 (57.8)	410 (57.7)	1181 (57.9)	516 (58.9)	329 (55.0)	533 (58.4)
	Unknown	18 (0.4)	0 (0.0)	14 (0.7)	4 (0.5)	0 (0.0)	0 (0.0)
Antibiotic before admission	Yes	743 (14.5)	62 (8.7)	313 (15.4)	24 (2.7)	85 (14.2)	259 (28.4)
	No	2972 (57.9)	581 (81.8)	622 (30.5)	834 (95.2)	329 (55.0)	606 (66.4)
	Unknown	1419 (27.6)	67 (9.4)	1103 (54.1)	18 (2.1)	184 (30.8)	47 (5.2)
Outcome diagnosis	Meningitis	363 (7.1)	107 (15.1)	87 (4.3)	42 (4.8)	22 (3.7)	105 (11.5)
	Pneumonia	72 (1.4)	37 (5.2)	11 (0.5)	4 (0.5)	12 (2.0)	8 (0.9)
	Septicemia	235 (4.6)	26 (3.7)	101 (5.0)	4 (0.5)	34 (5.7)	70 (7.7)
	Other/multiple	1022 (19.9)	301 (42.4)	120 (5.9)	173 (19.7)	255 (42.6)	173 (19.0)
	Unknown	3442 (67.0)	239 (33.7)	1719 (84.3)	653 (74.5)	275 (46.0)	556 (61.0)
Outcome	Discharged Alive	3107 (60.5)	561 (79.0)	935 (45.9)	308 (35.2)	456 (76.3)	847 (92.9)
	Died	278 (5.4)	93 (13.1)	49 (2.4)	52 (5.9)	39 (6.5)	45 (4.9)
	Unknown	1749 (34.1)	56 (7.9)	1054 (51.7)	516 (58.9)	103 (17.2)	20 (2.2)
Total no. of suspected cases recruited^f^		5134 (100.0)	710 (13.8)	2038 (39.7)	876 (17.1)	598 (11.6)	912 (17.8)

^a^Abubakar Tafawa Balewa University Teaching Hospital.

^b^Lagos University Teaching Hospital.

^c^University of Benin Teaching Hospital.

^d^University of Ilorin Teaching Hospital.

^e^University of Nigeria Teaching Hospital.

^f^Suspected cases include cases that were defined as probable per World Health Organization case definition guidelines [16].

The clinical characteristics of the children enrolled are detailed in Table 2. In summary, 75 (2.2%: 75/3338) children who presented with clear CSF samples had confirmed bacterial meningitis. However, a higher proportion of patients (4.5%: 75/1667) with turbid, xanthrochromic and blood-stained CSF samples had confirmed bacterial meningitis. The percentage of patients with PBM increased with WBC count; 1.7% (67/3888) of patients with a low WBC count (≤10 cells/mm^3^) had bacterial meningitis, whereas 3.4% (12/352) and 14.8% (29/196) of children with >10 to 100 cells/mm^3^ and >100 cells/mm^3^, respectively, were infected. More patients with PBM had high levels of protein (>100 mg/dL) in their CSF (9.5%: 42/441) compared to those with low protein levels ≤100 mg/dL (2.7%: 81/3010). However, more children with CSF glucose levels of ≤40 g/dL had PBM (4.5%: 57/1279) compared to 2.9% (64/2193) of patients with lower glucose levels (>40 g/dL).

**Table 2.** Summary of Clinical Characteristics of Patients in Relation to Causative

		Recruited	Tested	Pneumococcus	Meningococcus	*Haemophilus influenzae*
Characteristic		n	n (%)	n (%)	n (%)	n (%)
Cerebrospinal fluid appearance	Clear	3338	3281 (98.3)	39 (1.2)	18 (0.5)	18 (0.5)
	Turbid	418	411 (98.3)	20 (4.9)	18 (4.4)	6 (1.5)
	Xanthrochromic	635	624 (98.3)	6 (1.0)	9 (1.4)	1 (0.2)
	Blood stained	614	607 (98.9)	4 (0.7)	8 (1.3)	3 (0.5)
	Unknown	129	85 (65.9)	2 (2.4)	0	1 (1.2)
White blood cell count (cells/mm^3^)	≤10	3888	3840 (98.8)	33 (0.9)	20 (0.5)	14 (0.4)
	>10 to 100	352	348 (98.3)	4 (1.1)	6 (1.7)	2 (0.6)
	>100	196	195 (99.5)	15 (7.7)	11 (5.6)	3 (1.5)
	Unknown/not done	698	625 (90.0)	19 (3.0)	16 (2.6)	10 (1.6)
Protein (mg/dL)	≤100	3010	2952 (98.1)	41 (1.4)	20 (0.7)	20 (0.7)
	>100	441	432 (98.0)	15 (3.5)	23 (5.3)	4 (0.9)
	Unknown/not done	1683	1624 (96.5)	15 (0.9)	10 (0.6)	5 (0.3)
Glucose (g/dL)	≤40	1279	1261 (98.6)	21 (1.7)	23 (1.8)	13 (1.0)
	≥40	2193	2146 (97.9)	35 (1.6)	19 (0.9)	10 (0.5)
	Unknown/not done	1662	1601 (96.3)	15 (0.9)	11 (0.7)	6 (0.4)
Total no. of suspected cases recruited^a^		5134	5008 (97.5)	71 (1.4)	53 (1.1)	29 (0.6)

^a^Suspected cases include cases that were defined as probable as per World Health Organization case definition guidelines [16].

There were a total of 153 (3.0%: 153/5134) cases of PBM observed at the 5 sentinel hospitals during the surveillance period in Nigeria. Pneumococcus was responsible for 71 cases (46.4%: 71/153) with a mortality rate of 14.1% (10/71). Meningococcal meningitis was confirmed in 53 (34.6%:53/153) pediatric patients with a case fatality rate of 20.7% (11/53). A total of 29 (19.0%: 29/153) children had meningitis caused by *H. influenzae*, among these 2 patients died; mortality rate of 3.4% (2/29). The overall mortality rate for confirmed bacterial meningitis cases was 15% (23/153).

### Distribution of Bacterial Pathogens

**Figure 1.**
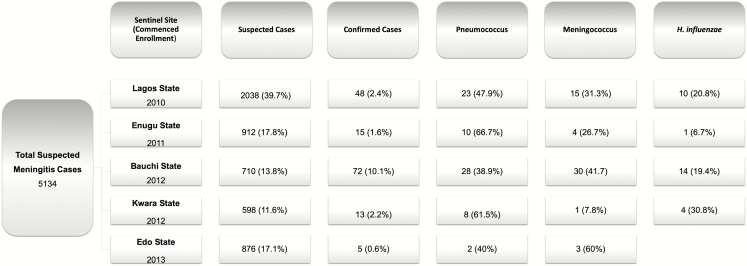
Distribution of suspected pediatric bacterial meningitis cases from 2010 to 2016 within 5 sentinel hospitals in 5 Nigerian states. A total of 5134 suspected pediatric bacterial meningitis cases were observed at 5 sentinel hospitals: Lagos University Teaching Hospital (Lagos State), University of Nigeria Teaching Hospital (Enugu State), Abubakar Tafawa Balewa University Teaching Hospital (Bauchi State), University of Ilorin Teaching Hospital (Kwara State), and University of Benin Teaching Hospital (Edo State). Each hospital commenced surveillance at different time points; however, surveillance continued for all hospitals until 2016. The number of suspected cases of bacterial meningitis and the number of confirmed cases of meningitis (World Health Organization definitions [16]) varied per hospital. The main causative agents for bacterial meningitis were *Streptococcus pneumoniae, Neisseria meningitidis,* and *Haemophilus influenzae.*

**Figure 2.**
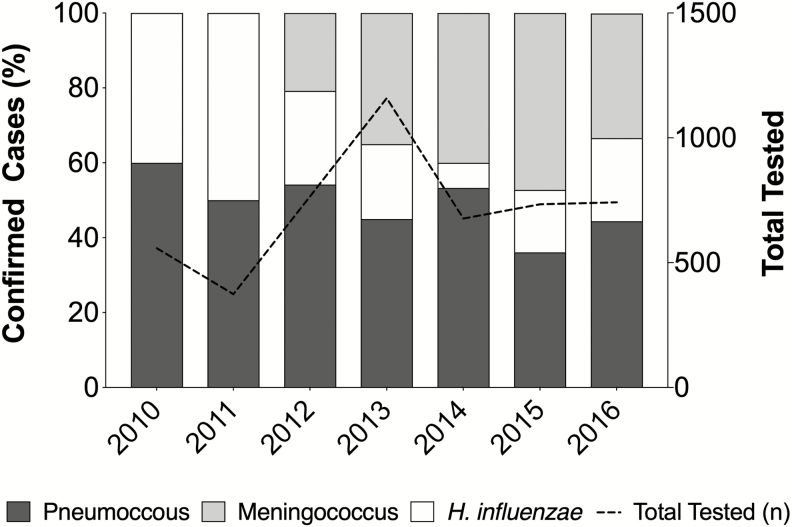
Proportion of confirmed pediatric bacterial meningitis cases and the pathogens responsible from 2010 to 2016 in 5 Nigerian states. The percentage of confirmed bacterial meningitis cases caused by *Streptococcus pneumoniae, Neisseria meningitidis,* and *Haemophilus influenzae* in children <5 years across 5 Nigerian states. A dashed black line indicates the total number of cerebrospinal fluid samples that were tested each year of surveillance.

The number of suspected PBM cases observed in each of the 5 sentinel hospitals varied annually with an average of 715.4 cases per year (Figure 1). In 2013, with 5 sentinel hospitals enrolled, the total number of suspected cases peaked at 1204. Lagos State had the highest proportion of suspected meningitis over the study period (39.7%: 2038/5134) and Kwara State the least (11.6%: 598/5134). Additionally, the prevalence of meningitis caused by each of the 3 bacterial pathogens varied annually (Figure 2).

**Figure 3.**
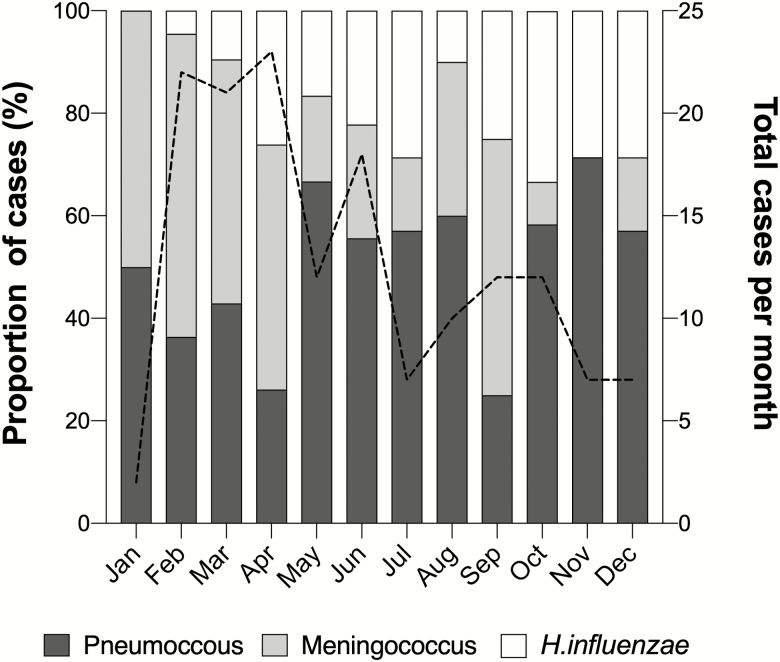
Monthly distribution of suspected pediatric bacterial meningitis cases for the period 2010 to 2016 within 5 sentinel hospitals across Nigeria. The percentage of pediatric bacterial meningitis (PBM) cases seen across 5 hospitals in 5 Nigerian states, caused by *Streptococcus pneumoniae, Neisseria meningitidis,* and *Haemophilus influenzae* per month. A black dashed line indicates the total number of PBM cases per month throughout the surveillance period.

The frequency of confirmed meningitis cases observed fluctuated between months of the year (Figure 3). Presentation of PBM was highest from February to June throughout the surveillance period, and the highest number of cases was recorded in April. The prevalence of each causative agent varied from month to month. For instance, from February to April, most cases were caused by meningococcus 34 (51.5%: 34/66) and from May to August the dominant pathogen switched to pneumococcus with 28 (59.6%: 28/47) cases.

### Serotype and Serogroup Distribution

Serotype analysis was attempted on 31 (43.7%: 31/71) pneumococcal positive CSF samples, however, 3 samples had a low concentration of DNA with CT values of >32 so could not be serotyped. Of the 28 isolates that were successfully serotyped, a variety of pneumococcal serotypes were responsible for PBM from year to year. For instance, the pneumococcal meningitis cases in 2011 and 2012 were caused by serotype 23F and 14, respectively, both of which are targeted by the PCV10 vaccine. We successfully serotyped 2 cases in 2013, which were caused by serotype 23F and 19A, which is a non-PCV10 serotype. In 2014, 10 pneumococcal meningitis cases were serotyped; of these, 1 case was caused by serotype 4, a PCV10 serotype. However, the remaining 9 (90%: 9/10) were caused by pneumococcal strains that were nontypeable by PCR and, thus, not covered by the current formulations of PCV. The 3 serotyped cases in 2015 were all caused by PCV10 serotypes, serotype’s 1, 5, and 18C. A further 11 cases were serotyped in 2016: 6 cases were caused by 4 PCV10 serotypes (4, 6B, 19F, and 23F), 4 cases were caused by pneumococci that were nontypeable by PCR, and 1 case was caused by serotype 23A, a non-PCV10 pathogen. Overall, half (50.0%: 14/28) of the pneumococcal meningitis cases that were successfully serotyped were caused by serotypes that are not targeted by PCV10 vaccine.

Furthermore, most of the meningococcal meningitis cases were reported from 2014 to 2016, with 41 (77.4%: 41/53) patients across the 3 years and a peak of 17 cases in 2015. In total, 20 (37.7%: 20/53) meningococcal isolates underwent serogrouping analysis. Four serogroups were observed within the surveillance period, the most prevalent was serogroup W, with 8 cases (40%: 8/20) reported in 2015, followed by a nongroupable strain causing 1 case in 2015 and 5 cases in 2016 (30%: 6/20). Serogroup B meningococcus was responsible for 5 case, 1 each in 2013 and 2015 and a further 3 in 2016. However, serogroup C was identified in 1 case of meningococcal meningitis in 2014.

Finally, of the 29 *H. influenzae* positive CSF samples, serotype analysis was successfully performed on 16 (55.2%: 16/29) of these. The most prevalent serotype was Hib, responsible for 1 case in 2012, 2 cases in 2013, and 8 cases in 2016 (69%:11/16). Hia caused 3 infections in 2015, and Hic was responsible for 2 infection in 2015. Interestingly, 2 of the patients with *H. influenzae* meningitis reported receiving at least 1 dose of the Hib vaccine, 1 case was caused by serotype Hia, and the other was not serotyped.

## DISCUSSION

We conducted detailed analysis of longitudinal PBM surveillance data from 5 hospitals in 5 states of Nigeria. We found the highest number of suspected bacterial meningitis cases in children from the youngest age group and a lower number of cases reported in children aged 12–23 months but rising in the eldest cohort of children aged 24–59 months (Table 1). This was unexpected, as previous studies in The Gambia, Oman, and Turkey have shown that the incidence of bacterial meningitis decreases with age, due to maturation of the immune response [23–25]. The mortality rate of suspected meningitis was 5.4% (278/5134). However, when excluding data for patients where the outcome at discharge was not recorded (34.1%: 1749/5134), the mortality rate increased to 8.2% (278/3385), suggesting that suspected meningitis patients were severely unwell. Sequelae was only reported for 2% (102/5134) of patients with suspected meningitis; however, this prevalence is likely to be higher given the large number of patients (62.3%: 3245/5134) without follow-up after discharge.

Diagnosis of acute bacterial meningitis can be difficult in sub-Saharan Africa where resources are limited and clinical features are similar to malarial infections [26]. Analysis of CSF through microbiology culture is the gold standard technique used to detect PBM; however, studies have found low bacterial recovery rates in West Africa [27]. Additionally, 14.5% (743/5134) of patients with suspected meningitis in Nigeria received antibiotics before admission, which may have contributed to low bacteriological recovery. Moreover, without the sophisticated laboratory techniques used, diagnosis of bacterial meningitis would have relied on clinical characteristics. Thus, the 44 (28.8%: 44/153) PBM patients presenting with clear CSF and low WBC count may have been misdiagnosed. A history of fever (84.3%: 129 /153) and presence of seizures (71.2%: 109/153) were the most common symptoms associated with confirmed PBM cases in Nigeria. However, an altered consciousness is a more accurate indicator of bacterial meningitis when combined with other common symptoms [28]. During surveillance, only 22% (34/153) of meningitis patients had an altered consciousness. Additionally, meningismus (10.5%: 16/153) and bulging fontanelle (8.5%: 13/153) were observed less frequently, highlighting the importance of considering all clinical symptoms when diagnosing PBM as outlined in the WHO guidelines [16].

West Africa has a bimodal climate: wet season occurs from mid-April until mid-October, and the remaining months are characterized by dry season. Available data suggest the intensity of bacterial meningitis epidemics are associated with the Harmattan winds during dry season; thus, epidemics are rare during rainy season [29, 30]. The seasonal distribution of PBM within Nigeria from 2010 to 2016 is in line with this trend (Figure 3). Previous studies have shown that respiratory syncytial virus (RSV) is also seasonal in Nigeria, with cases mainly observed during the dry season and often peaking in November [31, 32]. Conversely, influenza has been shown to have year-round activity within Nigeria [33, 34]. Moreover, the seasonality of malaria transmission within Nigeria differs across the country based on the various ecological regions. Thus, malaria transmission is often year-round in southern Nigeria where there are high levels of mangrove swamps but lasts 3 months or less in northern regions such as the Sahel-savannah [35, 36].

Vaccines targeting the main bacterial pathogens causing meningitis are effective at reducing morbidity and mortality [37, 38]. The Hib vaccine was introduced in phases across Nigeria from May 2012 to May 2014, and 41.4% (12/29) of the *H. influenzae* associated meningitis cases occurred during this period, with a further 48.3% (14/29) cases postvaccine introduction. We identified 11 cases of Hib meningitis; of these, 7 patients had not received the Hib vaccine, and the Hib vaccination status for the remaining 4 patients was unknown. The WHO and UNICEF 2017 national immunization coverage estimates for the Hib containing vaccine in Nigeria were <50% [15]. Additionally, in 2017, Nigeria’s National Primary Health Care Development Agency (NPHCDA) found that only Bauchi and Kwara of the 5 states participating in PBM surveillance reported >50% coverage of the Hib vaccine, indicating that coverage across Nigeria needs to be improved [39].

MenAfriVac against serogroup A has not been introduced into routine immunization programs in Nigeria but was given to at risk populations during mass campaigns from 2011 to 2014 in high-risk states. Therefore, the recording of MenAfriVac vaccination status of patients during surveillance was limited, with 4 patients reporting they had received the vaccines, 650 patients reporting they had not, and 4480 patients where this information was unknown or not recorded. However, of the 20 isolates we serogrouped, none were serogroup A; instead, serogroup W was the predominant strain, followed by nongroupable strains. Expansion of nonvaccine serogroups due to vaccine selection pressures could be responsible for the increased incidence of nonserogroup A meningococcus, but, as limited serogrouping data were obtained, further work is required to conclude serotype replacement is occurring [40].

Pneumococcus was the predominant pathogen causing 71 (46.4%: 71/153) PBM cases during surveillance in Nigeria. A high incidence of pneumococcal meningitis is also common in The Gambia [25]. Of the 5 states where surveillance occurred, Edo state commenced PCV10 vaccinations in 2014, and only 2 pneumococcal meningitis cases were reported here afterward. The remaining 4 states received PCV10 in 2016; thus, pre- and postvaccine comparisons are not possible, and we were unable to record the PCV10 vaccination status of patients throughout our surveillance period. However, 46.4% (13/28) of the isolates serotyped were strains that are included in PCV10 and thus could have been prevented by immunization. The 2017 WHO and UNICEF national immunization coverage estimates suggest that coverage rates for PCV10 are <50% in Nigeria [15]. For each of the 5 states enrolled in surveillance, coverage of 3 PCV10 doses from January to November 2017 was >50% in Bauchi and Kwara only [39]. We found 1 case of pneumococcal meningitis caused by serotype 19A, targeted by PCV13 but not PCV10, and 13 cases caused by pneumococcal serotypes that were nontypeable and thus not covered by current PCV formulations. Serotype replacement of vaccine serotypes with nonvaccine serotypes is a phenomenon that has been widely reported since the introduction of PCVs [41, 42]. However, due to the limited pneumococcal serotype data reported and the recent introduction of PCV10, surveillance should be continued in Nigeria to monitor the burden of vaccine preventable bacterial meningitis and any potential changes in serotype distribution over time.

### Limitations

During this surveillance, children with suspected meningitis were not followed up after hospital discharge. There were a total of 278 (5.4%: 278/5134) in-hospital deaths; of these, 23 (8.2%: 23/278) were patients with confirmed bacterial meningitis. However, the outcome for 1749 patients (34.1% 1749/5134) was unknown or not recorded, and of these, 26 (1.5%: 26/1749) were patients with confirmed bacterial meningitis. Therefore, the case fatality of 5.4% (278/5134) among children with suspected meningitis may have been higher, but this cannot be confirmed. Additionally, 102 (2%: 102/5134) patients were reported to have long-term sequelae; however, 3245 (63.2%: 3245/5134) were not followed up after discharge, and the HIV status was not routinely recorded during the surveillance. Moreover, there were discrepancies between the level of reporting from each sentinel hospital. In future studies, it would be beneficial to record information regarding long-term sequelae, outcome at discharge, and HIV status for all patients with suspected meningitis and to do this in a standardized manner across all sites. This would allow more accurate rates of morbidity and mortality associated with bacterial meningitis within the surveillance populations to be estimated.

## CONCLUSIONS

Pneumococcus was responsible for the majority of PBM cases in Nigeria; however, PCV10 has now been introduced within all states included in surveillance, and with improved vaccine coverage it is expected that pneumococcal meningitis rates will start to decline. Hib remains responsible for a significant proportion of meningitis despite vaccine introduction. Therefore, our findings emphasize the need for further monitoring to establish the impact of conjugate vaccines on reducing the prevalence of bacterial meningitis within Nigeria. Further serotype/group data for PBM cases is required to understand the distribution of specific pathogen strains across Nigeria and to enhance efficacy of target vaccines.

## Notes

***Invasive Bacterial Disease*** ***Writing Group members.*** Brenda Kwambana Adams, Senghore Madikay, Effua Usuf, Archibald Worwui, Uzochukwu Egere, Akram Zaman, Catherine Okoi, Florian Gehre, Leopold Tientcheu, Nuredin Ibrahim Mohammed, Felix Dube, Peter Ndow, Sambou M. Suso, Sheikh Jarju, Dam Khan, Chinelo Ebruke, Rowan Bancroft, Jason M. Mwenda, and Martin Antonio.

***Author contributions***. M. A. and J. M. M. established the World Health Organization Regional Office for Africa–supported Paediatric Bacterial Meningitis Surveillance Network in West Africa. M. A. supervised the overall network including setting up the sentinel surveillance system. B. N. T., I. F., M. B. A., M. F. B., O. P. O., A. H. I., N. M. L., B. O. E., N. O., C. J. I., F. N., U. C. O., F. U., A. O. S., A. F., S. A. A., J. A., A. G. Y., P. O., C. K., V. E., and O. J. M. clinically investigated and recruited the patients at the sentinel sites, collected demographic data, performed microbiological testing, and shipped cerebrospinal fluid and bacterial isolates to the Medical Research Council (MRC) Unit The Gambia (MRCG) for confirmatory testing and molecular analysis supervised by B. K. A. and M. A. B. K. A. and M. A. developed the analysis plan and contributed to analysis and interpretation of data along with the IBD writing group. R. E. B., B. K. A., and M. A. drafted the article along with B. N. T. All authors contributed to the interpretation of the findings and the writing of the final article.

***Acknowledgments.*** The authors thank the WHO Country Office of Nigeria and WHO Inter-country Support Team for coordination, advice and support throughout the project. They thank the surveillance team members as well as patients and their families in Nigeria; the staff and students at the MRCG at London School of Hygiene and Tropical Medicine [LSHTM], as well as the IBD writing group for their advice and input.

***Financial support.*** Financial support for sentinel site surveillance was provided by the Federal Ministry of Health Nigeria, GAVI - the Vaccine Alliance, through a grant to the WHO for the African Paediatric Bacterial Meningitis Surveillance Network.

***Supplement sponsorship.* This supplement was supported with funds from Gavi, the Vaccine Alliance through The World Health Organization and the CDC Foundation, and The Medical Research Council Unit The Gambia at the London School of Hygiene and Tropical Medicine.**

***Potential conflicts of interest.*** All authors: No reported conflicts of interest. All authors have submitted the ICMJE Form for Disclosure of Potential Conflicts of Interest. Conflicts that the editors consider relevant to the content of the manuscript have been disclosed.
