# Erratum

**DOI:** 10.1093/cid/ciz1222

**Published:** 2020-01-14

The online publication of this article [Tagbo BN, Bancroft RE, Fajolu I et al. Pediatric Bacterial Meningitis Surveillance in Nigeria From 2010 to 2016, Prior to and During the Phased Introduction of the 10-Valent Pneumococcal Conjugate Vaccine. Clin Infect Dis. https://doi.org/10.1093/cid/ciz474] was inadvertently listed with Volume 69, Issue Supplement 3, resulting in an incorrect citation line. The article is now listed in the correct issue online, and the citation line has since been corrected to “*Clinical Infectious Diseases*, Volume 69, Issue Supplement_2, 15 September 2019, Pages S81–S88, https://doi.org/10.1093/cid/ciz474.”

The publisher regrets this error.

